# Adaptive walking performance is related to the hip joint position sense during active hip flexion rather than during passive hip flexion

**DOI:** 10.3389/fspor.2025.1510447

**Published:** 2025-02-13

**Authors:** Taishi Matsui, Kosuke Hirata, Naokazu Miyamoto, Ryota Akagi

**Affiliations:** ^1^ Graduate School of Engineering and Science, Shibaura Institute of Technology, Saitama, Japan; ^2^ Institute of Health and Sport Sciences, University of Tsukuba, Tsukuba-shi, Ibaraki, Japan; ^3^ Faculty of Health and Sports Science, Juntendo University, Inzai, Chiba, Japan; ^4^ Institute of Health and Sports Science & Medicine, Juntendo University, Inzai, Chiba, Japan; ^5^ College of Systems Engineering and Science, Shibaura Institute of Technology, Saitama, Japan

**Keywords:** crossing over obstacles, angular velocity, error from the target angle, isotonic contraction, dynamometer

## Abstract

The purpose of this study was to investigate the relationship between hip joint position sense during active or passive hip flexion and adaptive walking performance across obstacles. After screening, 30 young men with the right dominant leg (age, 21 ± 2 years) participated in the experiment. To measure adaptive walking performance on the first day, the participants stepped over an obstacle underfoot with the left leg just high enough to avoid touching the obstacle. The difference between the height of the knee joint at the moment of crossing the obstacle and the height of the obstacle was normalized to the lower limb length and used to evaluate performance. To measure hip joint position sense on the second day, the participants adjusted their left hip joint angle to the target angle (range of joint motion: 80° of hip flexion) by active or passive hip flexion using a dynamometer. Although the absolute error in hip joint position sense during active hip flexion (6.3° ± 4.4°) significantly correlated with that during passive hip flexion (23.2° ± 11.0°) (*r* = 0.507, *P* < 0.001), a notable difference was observed between the two (*P* < 0.001). The normalized knee joint height was significantly correlated with the absolute error of hip joint position sense during active hip flexion (*r* = 0.477, *P* < 0.001) but not during passive hip flexion. The results of this study suggest a strong association between hip joint position sense under conditions that closely resemble actual walking behavior and adaptive walking performance, such as crossing over obstacles.

## 1 Introduction

Proprioception is the conscious perception of the state of one's own limbs and trunk based on afferent signals generated by the peripheral nervous system (1). Joint position sense, which is one of the three main senses that comprise proprioception and is the sense that recognizes the position and joint angle of each body part, has a significant influence on actual physical movements. For instance, previous studies have shown that the better the hip and knee joint position sense, the better the adaptive walking performance in precisely aligning the foot of the lead limb to the target height (2–4). When walking across an obstacle by precisely controlling the trajectory of the foot according to the height of the obstacle, joint position sense, visual inputs and efference copy are integrated into the central nervous system, and joint movements are appropriately controlled as outputs based on these inputs (3, 5, 6). Here, efference copy refers to the internal duplication of motor command signals sent from the central brain to peripheral muscles, which is simultaneously conveyed to sensory brain regions along with afferent proprioceptive inputs (7–9). On the other hand, normal participants can reduce errors with obstacles and accurately cross obstacles with a small clearance height through repeated practice, even in a walking task, when wearing goggles that block the lower half of the visual field (10–13). This observation suggests that joint position sense and efference copy play a more critical role in adaptive walking performance than visual inputs. To accurately assess joint position sense in conjunction with efference copy, it is essential to evaluate joint position sense during active movements to investigate the determinants of adaptive walking performance; however, to date, joint position sense has been evaluated passively, and the evaluation of joint position sense under such conditions deviates greatly from the active joint movement in the actual walking motion.

The physiology of fusimotor drive involves the interaction between *γ*-motoneurons and intrafusal muscle fibers (14). Fusimotor drive, together with the aforementioned efference copy, is considered to enhance sensory function during active joint motion compared to passive joint motion, thereby improving the accuracy of joint position sense measurement (15, 16). This suggests that joint position sense during active joint movement is more accurate than that during passive joint movement. In adaptive walking, where the pedestrian barely steps over an obstacle, the hip joint must be flexed to an appropriate joint angle at an appropriate speed, and the foot must be pulled up to a height where it is not just barely caught by the obstacle. Therefore, it is crucial to measure hip position sense during active hip flexion to examine its relationship with adaptive walking performance. The purpose of this study was to investigate the relationship between hip joint position sense during active or passive hip flexion and adaptive walking performance. We hypothesized that a closer relationship exists between adaptive walking performance and hip joint position sense during active hip flexion than during passive hip flexion because of the more accurate hip joint position sense evaluated by active than by passive hip flexion.

## 2 Materials and methods

### 2.1 Participants

Prior to the experiment, the dominant and non-dominant legs of the 42 potential participants were verified using Waterloo Footedness Questionnaire Revised (17). The dominant leg was determined to be the right leg if the score was 5 or higher and the left leg if the score was −5 or lower. Owing to the convenience of setting up the experimental environment, only participants whose dominant leg was the right leg were selected. Consequently, 30 young men [age: 21 ± 2 years, height: 172.7 ± 5.7 cm, body mass: 64.0 ± 8.2 kg; mean ± standard deviation (SD)] participated in the experiment. None of the participants had a previous or current knee or hip joint injury. This study was approved by the Ethics Committee of Shibaura Institute of Technology (No. 22–029) and was conducted in accordance with the guidelines outlined in the Declaration of Helsinki. The participants were informed of the study's purpose and potential risks and provided written informed consent before participation.

### 2.2 Experimental procedures

**Figure 1:**
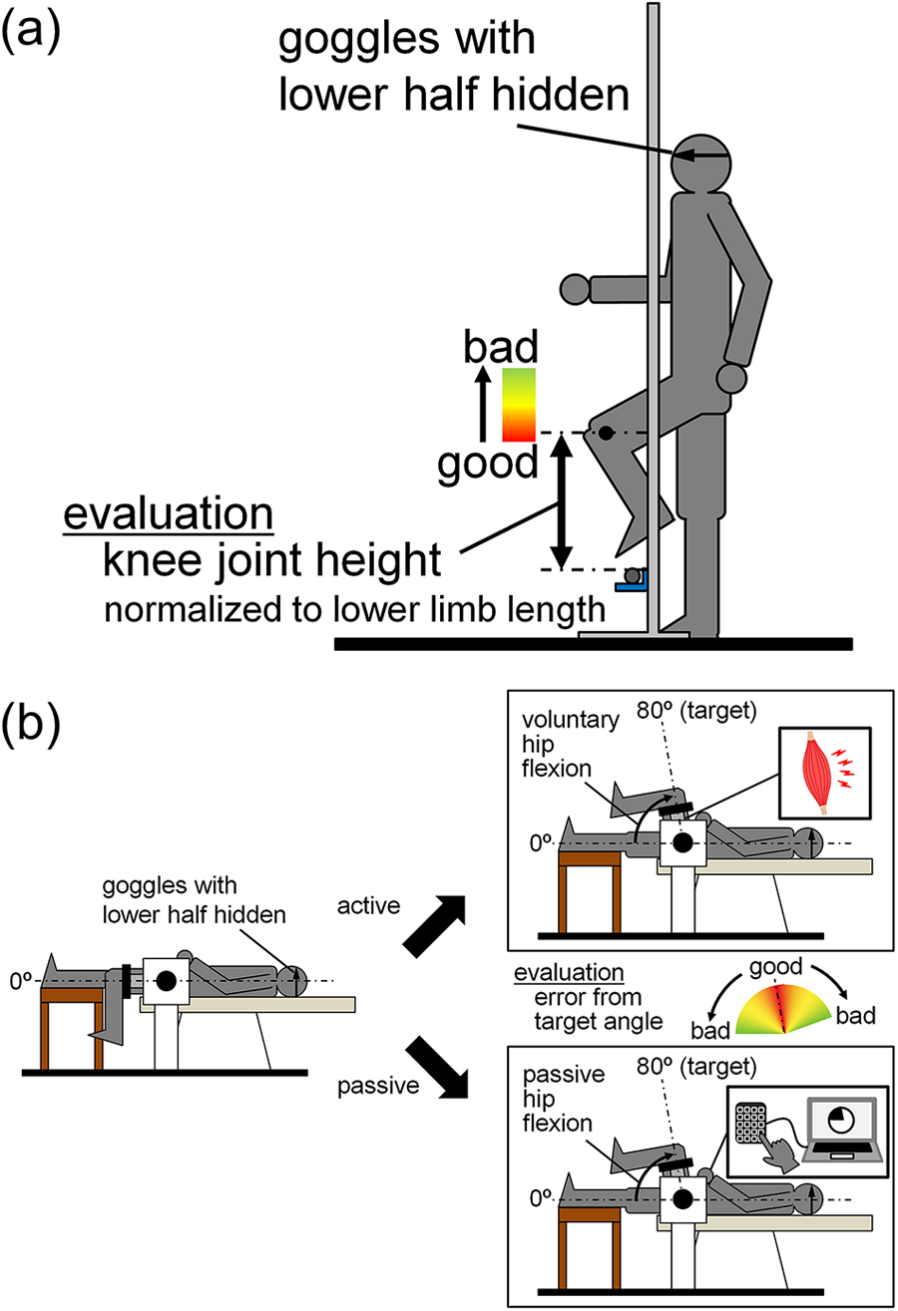
Experimental setup for measurements of adaptive walking performance **(a)** and hip joint position sense **(b)**.

Measurements were taken on the left leg (i.e., the non-dominant leg) on two separate days. Adaptive walking performance was determined on the first day, and hip joint position sense was measured on the second day. To measure adaptive walking performance, we employed a walking task in which participants were instructed to step over an obstacle underfoot with the left leg just high enough to avoid touching the obstacle (Figure 1a). To measure hip joint position sense, we employed a task in which the hip joint angle was adjusted to the target angle by active or passive hip flexion using a dynamometer (CON-TREX MJ; Physiomed, Schnaittach, Germany) (Figure 1b).

### 2.3 Measurement of adaptive walking performance

Before measuring adaptive walking performance, the position of the obstacle was set. The height from the ground to the center of the lateral malleolus of the participants was measured in 0.1 cm increments using a steel tape measure while the participants were in a static standing position. The thigh and lower leg lengths of the participants were also measured with a steel tape measure in 0.5 cm increments, with the participants in the same position. Two reference markers for motion analysis were applied to the lateral malleolus and lateral femoral epicondyle (one marker for each). A walking trial was then conducted to set the height of the obstacle using high jump stands and a 15-mm diameter round aluminum bar. After determining the appropriate starting position for the participants to walk over the bar with their left leg on the third step, the participants walked three steps from the starting position to the position where the obstacle was placed, lifting their feet as high as possible (Figure 1a). The participants were instructed to stop the movement at the position when they raised their foot highest on the third step, and the length from the ground to the center of the lateral malleolus of the left leg was measured to the nearest 0.1 cm using a stainless steel ruler. This trial was performed 10 times, and the following equation was used to determine the height of the top of the bar from the ground in the subsequent adaptive walking task:

$$\frac{1}{10}\overset{10}{\underset{i=1}{\sum }}\;\left\{\frac{4}{10}(w_{i}-s)+s\right\}$$

where *s* is the height from the ground to the center of the lateral malleolus in the static standing position, and *wi* is the length from the ground to the center of the lateral malleolus in the third step. By making this calculation, the height of the obstacle was adjusted between individuals to minimize the effect of individual differences in the hip joint range of motion and lower limb length (sum of the thigh and lower leg lengths) on the experimental results.

Adaptive walking performance was recorded using an iPad camera (iPad Air 5, Apple Inc., Cupertino, USA) positioned to capture the participants’ walking and obstacles from the sagittal plane at 240 fps. The participants wore goggles designed to obscure the lower half of their visual field, and were instructed to synchronize one step while walking with one beat of a metronome at 100 bpm. This speed was determined after preliminary experiments at several walking speeds, considering individual differences in height, lower limb length, and stride length. The first 10 walking trials were practice trials. After each trial, the participants checked the iPad screen to determine the degree of hip flexion and the positions of the knee joint and foot when the lateral ankle of the left leg passed directly over the bar (Figure 1a). Based on this confirmation, the participants attempted to adjust the height at which they raised their feet by flexing their hip joint in subsequent trials. The actual measurement trials were then repeated until ten successful trials were achieved. All trials in which the bar did not fall when the participants stepped over it were considered successful. After all the videos of the successful trials were copied to a personal computer, the height of the marker at the epicondyle from the ground at the moment the lateral ankle of the left leg passed directly over the bar was measured using ImageJ (ImageJ 1.52a, National Institutes of Health, Bethesda, USA) for each of the 10 trials. The difference between this height and the height of the obstacle (from the ground to the top of the bar) was then calculated. The smaller this difference was, the better the adaptive walking performance was judged to be. The difference between the height of the marker at the knee joint and the height of the bar was then calculated for each trial. The calculated values were then normalized to the lower limb length and used for performance evaluation.

### 2.4 Measurement of hip joint position sense

The participants were placed in the supine position in the reclining seat of the dynamometer [i.e., the hip joint angle was 0° flexed (anatomical position)]. This measurement posture differed from the posture during adaptive walking performance, owing to the constraints imposed by the dynamometer settings. The lower left leg was drooped, and the knee joint angle was approximately 90° flexed (anatomical position = 0°). The pelvis, torso, and thigh of the right leg were secured to the reclining seat of the dynamometer using non-elastic belts. Care was taken to adjust the centers of rotation of the hip joint and dynamometer. The lever arm of the dynamometer was attached to the distal part of the thigh of the left leg using a non-elastic strap. The dynamometer was connected to a 16-bit analog-to-digital converter (PowerLab16/35, ADInstruments, Bella Vista, Australia) and the data on the joint torque and the joint angle output from the dynamometer were recorded at a sampling frequency of 2 kHz on a personal computer using LabChart software (version 8.1.16, ADInstruments, Bella Vista, Australia).

To determine the resistance during the measurement of hip joint position sense with active hip flexion, the participants were asked to perform isometric maximal voluntary contraction (MVC) with the aforementioned hip and knee joint angles. After performing warm-up procedures, which comprise submaximal contractions, the 3-s isometric MVC of the hip flexor was repeated with a 1-min rest between each repetition until the difference between the highest and second-highest peak torque values was within 10%.

Hip joint position sense was initially assessed during passive hip flexion, followed by an assessment during active hip flexion. Both conditions included three practice trials, followed by 10 measured trials. In each trial, the starting hip joint angle was set at 0° of flexion, with the target hip joint angle set at 80° of flexion (i.e., the range of motion of the hip joint was set to 80°). Prior to each trial, the participants’ hip joints were passively moved to the target joint angle, and they were given approximately 30 s to memorize this position. During the measurements, participants wore the same goggles used in the adaptive walking performance to eliminate visual information regarding hip motion. The absolute error from the target angle in 10 measurements was determined. The smaller this error was, the better the joint position sense was.

In the measurement of hip joint position sense during passive hip flexion, the dynamometer was set up so that the hip joint was flexed at an angular velocity of 100°/s. The participants were instructed to hold a numeric keypad connected to the personal computer on which joint torque and joint angle were recorded, and press the enter key on the keypad as soon as they felt their hip joint angle had reached the target angle. To measure hip joint position sense during active hip flexion, the isotonic mode of the dynamometer (resistance: 10% of the highest value of isometric MVC torque) was used. The participants were instructed to flex their hip joints from 0° to 80° of hip flexion in approximately 0.6 s, synchronized to a 100 bpm metronome. This tempo was consistent with that used during the adaptive walking performance described earlier. Trials in which participants decelerated and re-accelerated during hip flexion, or transitioned from hip flexion to extension, were excluded from the analysis.

### 2.5 Statistical analyses

All statistical analyses were performed using two statistical analysis software programs (SPSS 28.0, IBM, USA; JASP version 0.19.2, University of Amsterdam, The Netherlands) and a spreadsheet (Excel 2016, Microsoft, USA). Statistical significance was set at *P* < 0.05.

The data on the absolute error of hip joint position sense, measured during active and passive hip flexion, as well as the normalized knee joint height during adaptive walking performance, were ranked in descending order of performance. A linear mixed model was employed to examine the differences between the absolute error of the hip joint position sense during active and passive hip flexion and to calculate the correlation coefficients for each combination of the three variables. The analysis utilized a total of 300 data sets, comprising 30 participants with 10 trials each. Before conducting the above statistical analyses, the Shapiro–Wilk test was performed on all variables. The results confirmed that the data for the absolute error of the hip joint position sense during active and passive hip flexion did not follow a normal distribution. Accordingly, all data were log-transformed prior to being subjected to the statistical analyses outlined above. For ease of interpretation, data in the text and figures are presented as means ± SDs of raw data.

## 3 Results

**Figure 2:**
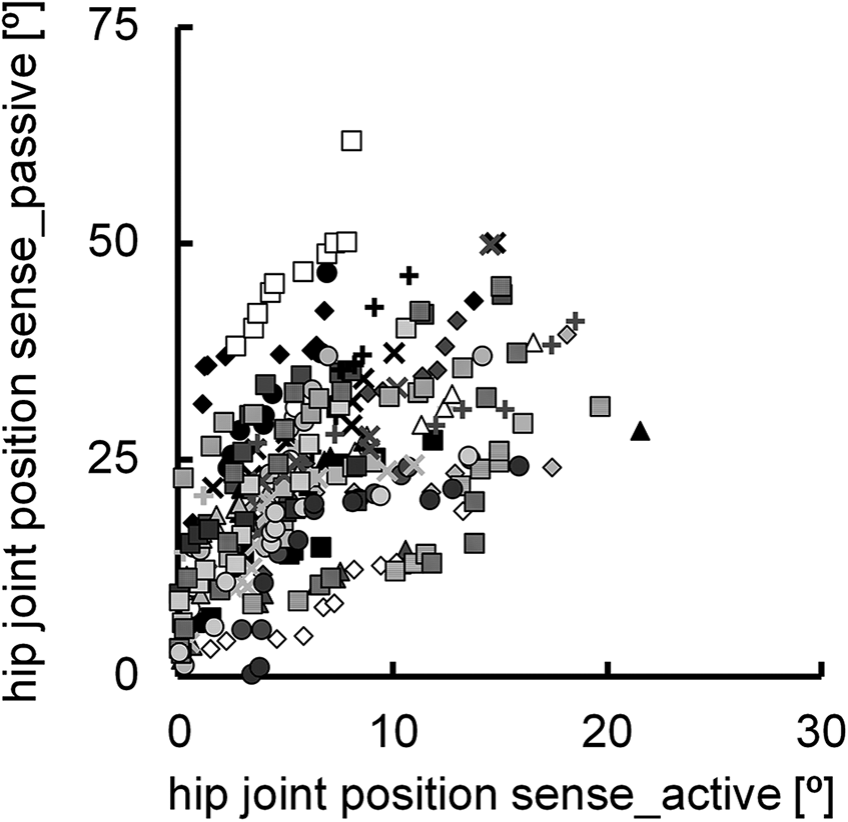
Scatter plots illustrating the relationship between the hip joint position sense during active and passive hip flexion (n = 300). Ten data points obtained from the same participant are indicated by the same symbol using raw data.

**Figure 3:**
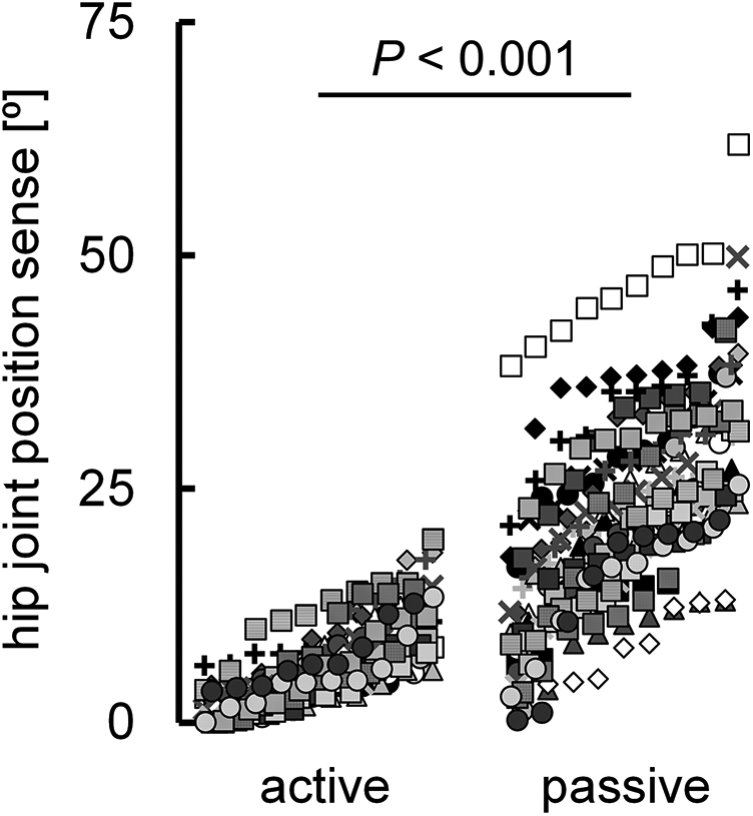
Diagram illustrating the differences in individual values between the absolute error of the hip joint position sense during active and passive hip flexion (*n* = 300). *P* value was calculated using log-transformed values for each variable. Ten data points obtained from the same participant are indicated by the same symbol using raw data.

**Figure 4:**
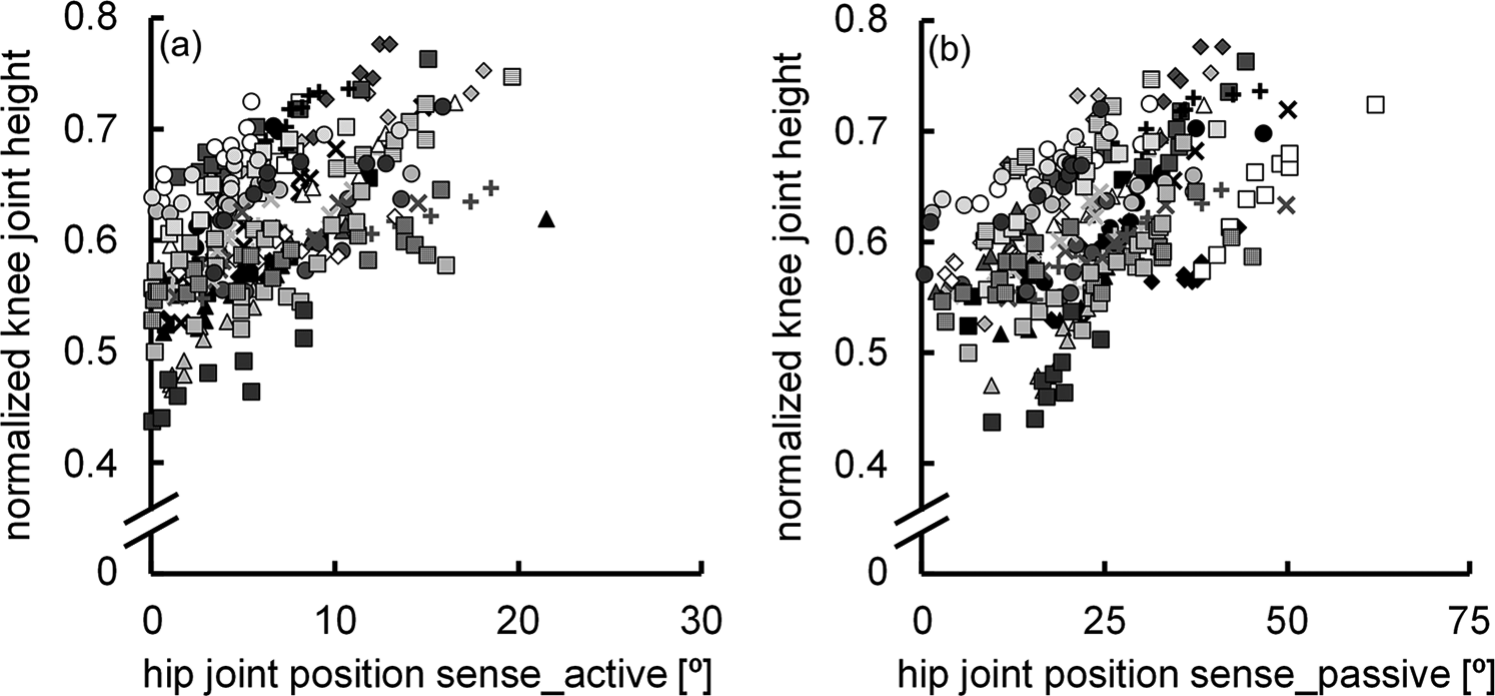
Scatter plots illustrating the relationships between hip joint position sense during active hip flexion and normalized knee joint height during adaptive walking performance **(a)**, and between hip joint position sense during passive hip flexion and normalized knee joint height during adaptive walking performance **(b)** (*n* = 300). Ten data points obtained from the same participant are indicated by the same symbol using raw data.

The normalized knee joint height during adaptive walking performance was 0.61 ± 0.07. The correlation coefficient between the hip joint position sense during active (6.3° ± 4.4°) and passive hip flexion (23.2° ± 11.0°) was significant (*r* = 0.507, *P* < 0.001) (Figure 2), with a notable difference between the two (*P* < 0.001) (Figure 3). The normalized knee joint height during adaptive walking performance correlated with the absolute error of the hip joint position sense during active hip flexion (*r* = 0.477, *P* < 0.001) (Figure 4a), indicating that those individuals with better joint position sense evaluated during active hip flexion are able to step over close to the height of the obstacle. On the other hand, the correlation coefficient between the absolute error of the hip joint position sense during passive hip flexion and the normalized knee joint height during adaptive walking performance could not be calculated because the coefficient of determination was found to be negative (*R*^2^ = −0.0813). (Figure 4b).

## 4 Discussion

### 4.1 Main findings

The main findings of this study were as follows: (1) the absolute error of the hip joint position sense was smaller during active hip flexion than during passive hip flexion, and (2) there was a closer association between the normalized knee joint height during adaptive walking performance and the absolute error of the hip joint position sense during active hip flexion than during passive hip flexion. These findings support the hypothesis of this study.

### 4.2 Importance of joint position sense evaluated during active joint movement

Although a significant correlation was observed between the absolute error of hip joint position sense during active (6.3° ± 4.4°) and passive hip flexion (23.2° ± 11.0°) (*r* = 0.507) (Figure 2), a notable difference was observed between the two (Figure 3). This finding is in agreement with previous studies that showed that joint position sense is better during active movement than during passive movement (15, 18–21). As described in the Introduction, fusimotor drive and efference copy are possible factors responsible for the difference in hip joint position sense during active and passive hip flexion. Muscle spindles, which are present in skeletal muscles, are proprioceptors that sense the length and speed of muscle stretching or contraction (16, 22, 23). The spindle was stretched when the muscle was stretched. The afferent firing rate also increased as the muscle spindle was stretched (16, 22). The state of the muscle spindle is regulated by fusimotor neurons, which play a crucial role in modulating and transmitting proprioceptive information regarding the transient position and movement of the associated extremity (24). These neurons are activated during joint movement. The active action of fusimotor neurons (i.e., the fusimotor drive described in the Introduction) adjusts the muscle spindle to an appropriate length, increases its sensitivity as a proprioceptor, and allows it to send more signals to the central nervous system (16). In this study, active hip flexion stretched the hip extensors, which are antagonist muscles. Given that muscle spindle afferent information from the lengthening antagonist muscle contributes to the related joint position sense during voluntary shortening of the agonist muscle (25, 26), the fusimotor drive in the hip extensors during active hip flexion may have improved hip joint position sense. On the other hand, as dscribed in the Introduction, efference copy—referring to information copied from centrifugal signals sent from the central brain to peripheral muscles as motor commands—is transmitted to sensory brain regions alongside afferent information from proprioception (7–9). Compared with passive joint movement, more information is obtained from the central nervous system during active joint movement, resulting in enhanced sensory function (5, 15).

In the present study, the normalized knee joint height during adaptive walking performance was correlated only with the absolute error of hip joint position sense during active hip flexion (Figures 4a,b). Generally, the amount of parameter variability affects the degree of correlation (27). The SD of the absolute error of hip joint position sense was 2.5 times greater during passive hip flexion (11.0°) than during active hip flexion (4.4°). Given the above, it is not surprising that the absolute error of hip joint position sense during passive hip flexion has a strong correlation with the normalized knee joint height during adaptive walking performance. Nevertheless, the observation that the normalized knee joint height during adaptive walking performance had a clear correlation with the absolute error of the hip joint position sense during active hip flexion but not with that during passive hip flexion highlights the strong influence of hip joint position sense during active hip flexion on adaptive walking performance. It has been reported that there is a relationship between athletes’ level of competition and their proprioceptive performance in active movements (28). However, to the best of our knowledge, no data have revealed the importance of joint position sense during active movement for a specific movement or the joint position sense associated with that movement. Based on the findings of this study, it is important to examine the relationship between joint position sense evaluated during active movements and performance in various single-joint and multi-joint movements. The adaptive walking performed in this study involves not only hip joint movements but also ankle and knee joint movements. With the rapid advancement of machine learning techniques for analyzing walking and running patterns (29, 30), it is likely that adaptive walking movements could be studied in greater detail than was possible in this study. These factors may have contributed to the slightly weakened correlation observed between the absolute error of hip joint position sense during active hip flexion and the normalized knee joint height during adaptive walking performance. Further research is needed to explore these aspects.

### 4.3 Influence of evaluation method of joint position sense on the interpretation of the present results

To the best of our knowledge, this is the first study to examine the relationship between joint position sense evaluated during active movements and performance in actual joint movements. Therefore, we discuss in detail the influence of the evaluation method of joint position sense adopted in this study on the results in terms of joint range of motion, speed of joint motion, and measurement posture during joint position sense evaluation.

In this study, the range of motion of the hip joint angle was considered to be as wide as 80°, assuming actual walking. This was a wider range of motion than that reported in previous studies that evaluated hip joint position sense (15°) (2–4). As mentioned above, the amount of change in muscle spindles is important in evaluating joint position sense. Large changes in joint angle and muscle length increase the amount of change in muscle spindles and the amount of signals sent to the central nervous system (16, 22, 23, 31). In addition to muscle spindles, cutaneous and joint receptors act as proprioceptors. Cutaneous receptors are similar to muscle spindles in their ability to sense skin stretch and contraction, as well as skin movement and direction (19, 23, 31, 32). Four types of receptors, Meissner corpuscles, Pacinian corpuscles, Merkel endings, and Ruffini endings, are responsible for the generation of proprioception (23, 33). When the skin is stimulated by stretching, these receptors respond and the afferents fire (33). The degree of activity of afferents innervating cutaneous receptors has been shown to be linearly related to the degree of skin stretching (34–36). Thus, for both muscle spindles and cutaneous receptors, the greater the change in joint angle, the greater the amount of signal sent to the central nervous system. Therefore, by evaluating joint position sense over a wide range of joint angles, it is expected that it will be easier to detect individual differences in the amount of signal sent to the central nervous system, and thus individual differences in joint position sense. Joint receptors also inform the central nervous system when a joint is approaching its anatomical limit angle (19, 23, 37). In fact, sensory function increases as joint position approaches the angular limit of joint range of motion (19, 38, 39). This suggests that it is important to have a wide range of joint motion and to set the starting and target joint angles to be very different when assessing joint position sense.

In this study, the participants were instructed to flex their hip joints using one beat on a 100 bpm metronome as a guide for angular velocity when measuring hip joint position sense during active hip flexion, and the hip joint angular velocity on the dynamometer was set at 100°/s during the measurement of hip joint position sense determined by passive hip flexion. Thus, under both conditions, the joint angular velocity was fast, consistent with adaptive walking. In other words, no multiple-joint angular velocities were set under both conditions. This is because previous studies have shown that the effect of joint angular velocity on the superiority or inferiority of joint position sense during both active and passive joint movements is small (40–42). Hence, it is suggested that the effect of joint angular velocity during the joint position sense evaluation adopted in this study had a small influence on the present results. In future studies, it is expected that there will be no major problems in evaluating joint position sense during active joint movements if the setting of the joint angular velocity is standardized among individuals.

In the present study, the measurement of hip joint position sense was first performed during passive hip flexion, considering that the measurement of joint position sense is negatively affected by muscle fatigue (43). As a result, it is possible that a learning effect influenced the measurement of hip joint position sense during active hip flexion. However, in the present study, a more than 3.5 times difference was observed between the absolute errors in hip joint position sense during active and passive hip flexion (6.3° vs. 23.2°; Figure 3). We believe that such a substantial difference cannot be solely attributed to the learning effect.

Owing to the limitations of the dynamometer setup, hip joint position sense was assessed in the supine position, which did not match the standing posture used in adaptive walking performance. In previous studies, the proprioception of the lower limb joints was reported to differ depending on the posture used in the measurement (44, 45). Therefore, the aforementioned discrepancy in measurement posture is a limitation of this study. However, since the postures at the time of evaluation were consistent between the participants, it can be inferred that this discrepancy had little influence on the present results.

This study exclusively included young males with a right-leg dominance. Future research should investigate whether similar trends observed in the present study are applicable to individuals with left-leg dominance, females, and individuals across various age groups, to evaluate the generalizability of the findings. Furthermore, it is important to consider the potential influence of confounding factors, such as participants’ physical activity levels, neuromuscular performance (e.g., reaction time), and proprioceptive training histories, which were not accounted for in the present study.

### 4.4 Conclusion

This study compared hip joint position sense during active hip flexion with that during passive hip flexion and investigated the relationship between hip joint position sense during active or passive hip flexion and adaptive walking performance. Hip joint position sense during active hip flexion was superior to that during passive hip flexion, and only the former correlated with adaptive walking performance. These results suggest a strong association between hip joint position sense under conditions that closely resemble actual walking behavior and the performance of adaptive walking, such as stepping over obstacles. A summary of our key findings is provided in the graphical abstract (Supplementary Figure S1), offering a visual representation of the study's conclusions.

## Data Availability

The raw data supporting the conclusions of this article will be made available by the authors, without undue reservation.
