# Supplementary figures and images for: Adaptive walking performance is related to the hip joint position sense during active hip flexion rather than during passive hip flexion
Source: Front Sports Act Living. 2025 Feb 13;7:1510447. doi: 10.3389/fspor.2025.1510447 (PMC11865228; doi:10.3389/fspor.2025.1510447)

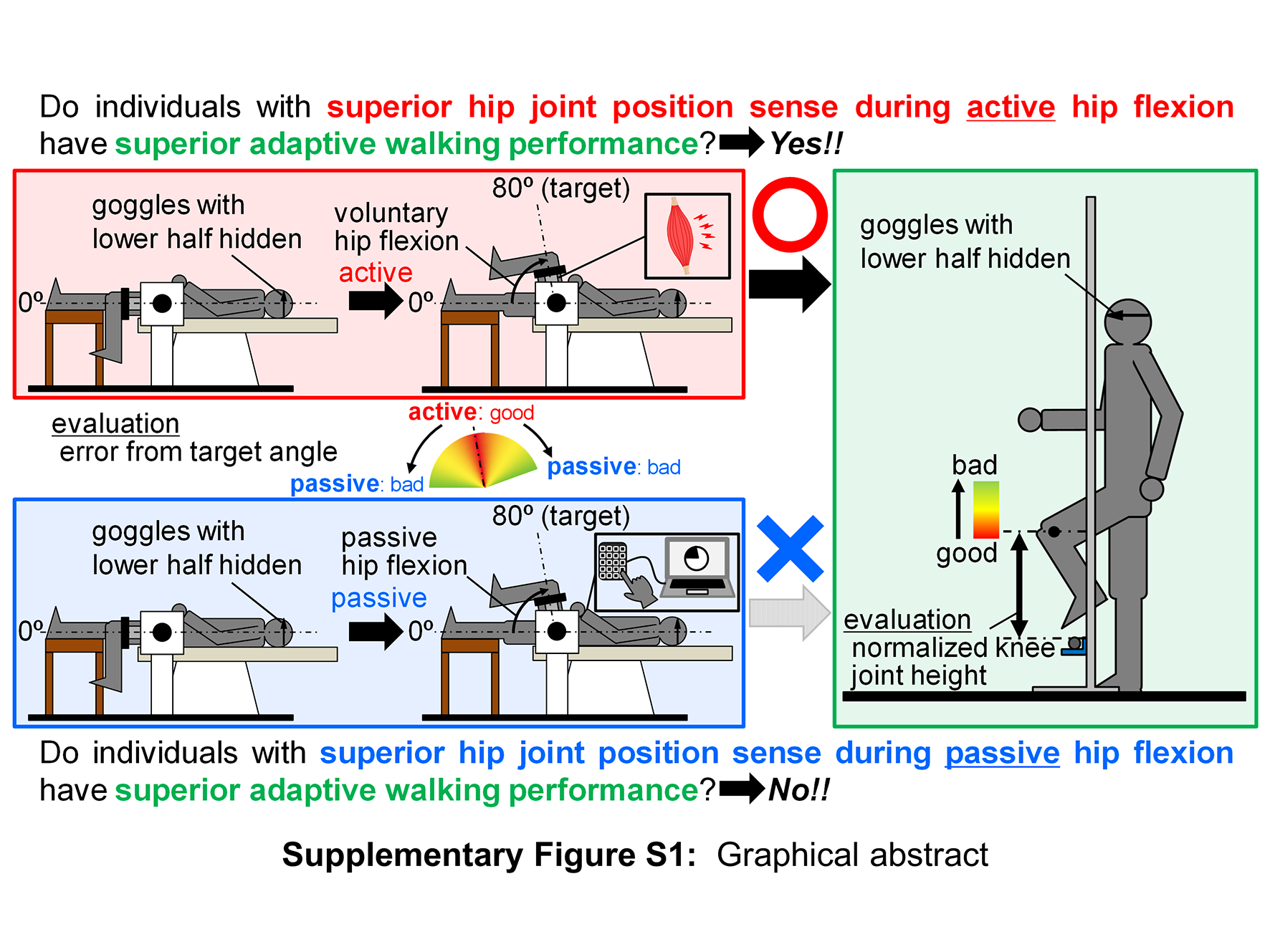

Supplement: Supplementary file 1 [file Image1.tif]
